# Improvement in precision grip force control with self-modulation of primary motor cortex during motor imagery

**DOI:** 10.3389/fnbeh.2015.00018

**Published:** 2015-02-13

**Authors:** Maria L. Blefari, James Sulzer, Marie-Claude Hepp-Reymond, Spyros Kollias, Roger Gassert

**Affiliations:** ^1^Rehabilitation Engineering Laboratory, Eidgenössische Technische Hochschule ZürichZurich, Switzerland; ^2^Chair in Non-Invasive Brain-Machine Interface, Center for Neuroprosthetics, École polytechnique fédérale de LausanneLausanne, Switzerland; ^3^Department of Mechanical Engineering, University of Texas at AustinAustin, TX, USA; ^4^Institute of Neuroinformatics, University of Zurich and Eidgenössische Technische Hochschule ZürichZurich, Switzerland; ^5^Neuroradiology Clinic, University Hospital ZurichZurich, Switzerland

**Keywords:** real-time fMRI, neurofeedback, motor imagery, motor skill

## Abstract

Motor imagery (MI) has shown effectiveness in enhancing motor performance. This may be due to the common neural mechanisms underlying MI and motor execution (ME). The main region of the ME network, the primary motor cortex (M1), has been consistently linked to motor performance. However, the activation of M1 during motor imagery is controversial, which may account for inconsistent rehabilitation therapy outcomes using MI. Here, we examined the relationship between contralateral M1 (cM1) activation during MI and changes in sensorimotor performance. To aid cM1 activity modulation during MI, we used real-time fMRI neurofeedback-guided MI based on cM1 hand area blood oxygen level dependent (BOLD) signal in healthy subjects, performing kinesthetic MI of pinching. We used multiple regression analysis to examine the correlation between cM1 BOLD signal and changes in motor performance during an isometric pinching task of those subjects who were able to activate cM1 during motor imagery. Activities in premotor and parietal regions were used as covariates. We found that cM1 activity was positively correlated to improvements in accuracy as well as overall performance improvements, whereas other regions in the sensorimotor network were not. The association between cM1 activation during MI with performance changes indicates that subjects with stronger cM1 activation during MI may benefit more from MI training, with implications toward targeted neurotherapy.

## Introduction

Motor imagery (MI) is a cognitive process in which individuals internally simulate a movement or action as being performed by themselves, but without any overt movement. MI is used in learning motor tasks, especially in sports, to complement physical training or to improve motor performance (Feltz and Landers, 1983; Alkadhi et al., 2005; Schuster et al., 2011 as review). It has been shown to enhance motor performance and learning in various tasks and over different time scales (Yàgüez et al., 1998; Mulder et al., 2004; Gentili et al., 2010) and even to increase muscle strength (Yue and Cole, 1992; Ranganathan et al., 2004). Furthermore, MI may prove valuable in situations where motor execution is impaired or abolished due to neurological disease, although its effect in neurorehabilitation has yielded mixed results (Malouin and Richards, 2013). This inconsistency is likely due to an incomplete understanding of the neural mechanisms underlying MI-based therapy, but also growing evidence that the neurological disorder itself may also interfere with MI ability (for review, see Di Rienzo et al., 2014). In this work, we aim to identify the role of contralateral primary motor cortex activity that may potentiate beneficial effects of MI on motor performance.

The central brain region in motor execution (ME) is the primary motor cortex (M1) for which structural and functional changes during learning have been reported (Dayan and Cohen, 2011; Hardwick et al., 2013). Motor imagery and motor execution are behaviorally closely related (Decety et al., 1989) and share similar neural networks (Jeannerod, 1994; Sharma and Baron, 2013). Numerous studies have shown an increase in excitability in contralateral M1 (cM1) during MI using transcranial magnetic stimulation (TMS, see Munzert et al., 2009 for review). Conversely, other brain imaging studies either did not find motor imagery activation in cM1 (Binkofski et al., 2000; Gerardin et al., 2000; Boecker et al., 2002; Naito et al., 2002) or reported a transient (Dechent et al., 2004) or weak involvement (Porro et al., 1996; Lacourse et al., 2005). In a recent brain imaging meta-analysis, Hétu et al. (2013) confirmed that MI in most studies activated a large number of primary and secondary motor areas in both hemispheres, including supplementary motor area (SMA), dorsal premotor cortex (PMd), as well as regions in the parietal lobe, basal ganglia and cerebellum. However, primary cortical activation was infrequent during MI (i.e., only 22% of the 75 experiments). This suggests strong inter-individual variability in MI ability (Guillot et al., 2008, 2009) and possibly differences in experimental procedures (Sharma et al., 2008), instructions given, imagery training length, level of motor expertise in the task to be imagined (Guillot and Collet, 2010), inability to objectively measure compliance (Sharma et al., 2006). All of these facets could explain the inconsistent outcomes of MI in neurorehabilitation (Malouin and Richards, 2013). Therefore, the neural underpinnings of MI have not yet been fully unraveled.

Instead of simply performing mental imagery, recent work has guided imagery via online feedback of metabolic correlates of neural activity from a desired brain region or network. This process is known as real-time functional magnetic resonance imaging neurofeedback (rtfMRI neurofeedback, for review see Sulzer et al., 2013a). Extracting the blood oxygen level dependent (BOLD) signal in a desired region-of-interest (ROI), rtfMRI neurofeedback has enabled self-regulation of cortical and subcortical brain areas (Ruiz et al., 2014). In the motor domain, experiments have repeatedly shown that rtfMRI-enhanced motor imagery can be used to successfully self-regulate primary and secondary sensorimotor areas (deCharms et al., 2004; Bray et al., 2007; Yoo et al., 2008; Zhao et al., 2013). As such, the use of neurofeedback can make activation of primary motor cortex more consistent during MI.

In addition to self-regulation, the evidence of causal brain-behavior relationships during neurally guided imagery further suggested the use of rtfMRI neurofeedback as a scientific tool (deCharms et al., 2005; Shibata et al., 2011; Scharnowski et al., 2012). For instance, over four training sessions, Bray et al. found improvements in reaction-time task in subjects who increased primary sensorimotor cortical activity (Bray et al., 2007), along with similar results in Parkinson's patients using feedback of SMA (Subramanian et al., 2011). More recently, self-regulation of dorsal premotor cortex led to improvements in motor sequence performance (Zhao et al., 2013). Taken together, these studies show that self-regulation of putative brain regions can result in appropriate behavioral changes in motor performance, but do not fully characterize the nature of these relationships.

Whereas previous experiments have shown that cM1 modulation during motor imagery affects motor performance, our goal was to characterize this relationship, hypothesizing a linear relationship between cM1 and motor performance changes. Here, rtfMRI neurofeedback is used as a tool to aid cM1 modulation during motor imagery toward this end. Therefore, we guided kinesthetic motor imagery (kMI) using feedback of cM1 activity and then associated the degree of modulation with control of force in a precision grip task. This study represents a novel approach toward identifying the neural correlates underlying the beneficial effects of motor imagery.

## Materials and methods

**Figure 1:**
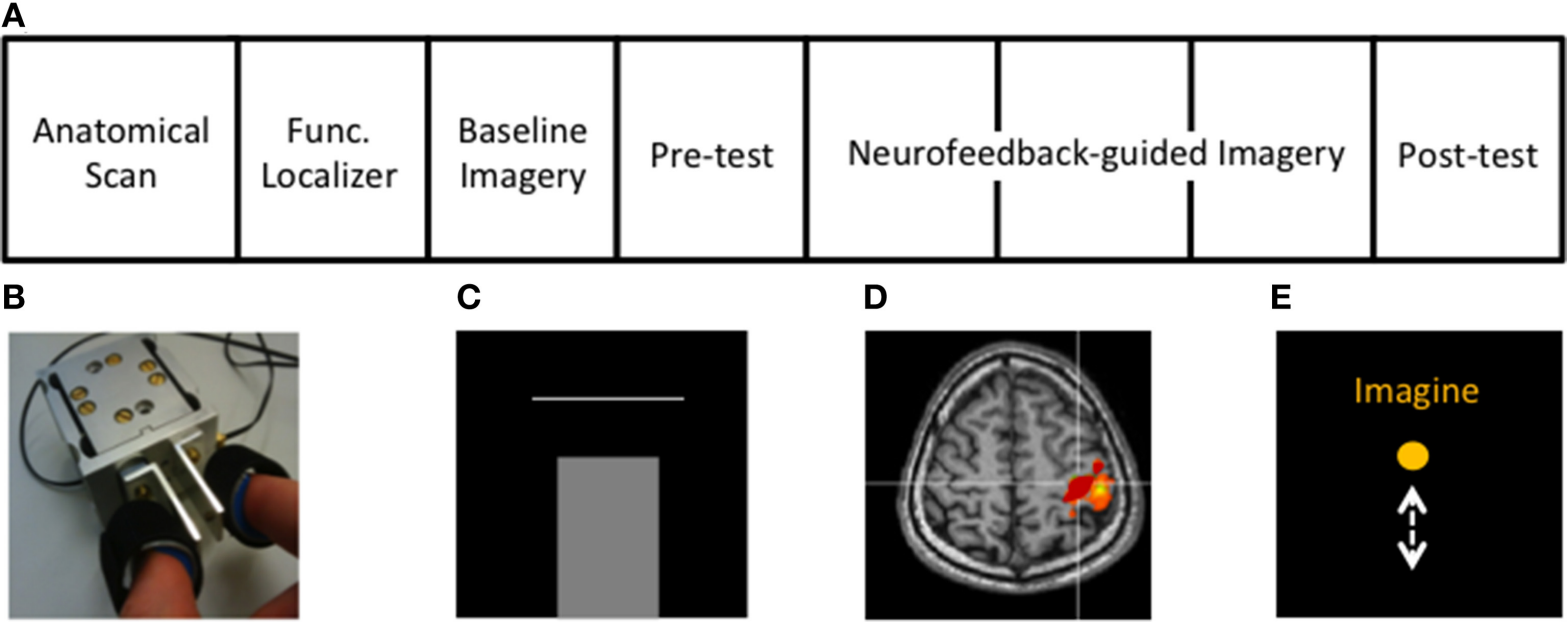
**Experimental protocol and setup. (A)** Structure of the rtfMRI session (see Methods). **(B)** Custom-built MR-compatible precision grip sensor used to perform precision grip both in the localizer and the isometric force target matching task, **(C)** Isometric force matching task in which the applied force (bar) has to match a horizontal line representing the target force (10 or 20% of MVF), **(D)** cM1 knob region (red) activated during the functional localizer, **(E)** Visual feedback displaying task instructions and a ball moving vertically during MI, proportional to the cM1 BOLD signal.

Fourteen healthy right-handed subjects (3 females) aged 24–32 years participated in a single fMRI experiment (1 day, see Figure 1A for protocol). One subject was excluded from the analysis due to failure to comply with the experimental instructions. The study was approved by the Zurich Cantonal Ethics Commission (KEK 2010-0190). After being informed on the safety regulations for an MR environment, all participants provided written consent.

### Motor imagery questionnaire

The Vividness of Movement Imagery Questionnaire (VMIQ, Isaac et al., 1986) was used to assess subjects' ability to perform motor imagery. The VMIQ includes 24 items, which can be grouped in six categories of four items, spanning from the imagery of basic (e.g., standing) to that of more complex movements (e.g., riding a bike). The questionnaire requires to imagine one item at a time from two different perspectives: (i) “watching somebody else” (external visual imagery) and (ii) “doing it yourself” (internal kinesthetic imagery). We asked our participants to perform only the kinesthetic part given our interest in kMI-based neurofeedback. For each item participants were required to rate the degree of clarity and vividness of the movement using a 5-point Likert scale. The scale ranges from 1 (*perfectly clear and vivid as normal vision*) to 5 (*no image at all*), thus a lower score indicated greater vividness.

### Familiarization on the force-matching task

Before the fMRI experiment the maximum voluntary grip force (MVF) of the participants' right dominant hand was measured, followed by the familiarization with the force-matching task outside the scanner. The motor task required the participants to move a vertically moving bar displayed on a screen between two horizontal target bars as quickly and accurately as possible, by exerting force on a MR-compatible precision grip force sensor (Gassert et al., 2008, Figure 1B). Participants then had to maintain the target force until the Release command was presented on the screen 2s after cue onset. The isometric grip force was either 10 or 20% of the subject's MVF, presented in a pseudorandom order. The gap between the bars narrowed as performance improved, i.e., 3 consecutive successful reaches resulted in a narrower gap level, and training continued until reaching a range of 5% of the respective target force. Visual feedback was created using custom-made software (Microsoft Visual Studio 2008, Redmond, WA). Force data were collected using a 12-bit data acquisition card (USB-6008, National Instruments, Austin, TX) sampled at 120 Hz. While performing the force-matching task, participants were asked to also focus on the motor and sensory aspects of the movement.

### Experimental procedure

The structure of the fMRI session is displayed in Figure 1A. First, the hand area of the contralateral primary motor cortex (cM1), i.e., left hemisphere, was localized using active isometric pinching and an anatomical overlay, i.e., with an anatomical and functional localizer (Figure 1D). Afterwards, baseline activity, without neurofeedback, in cM1 during kMI was acquired (baseline imagery). This was followed by an assessment of motor performance (behavioral pre-test) using a similar experimental protocol as that of the familiarization. The participants then performed neurofeedback-guided motor imagery of pinching followed by the behavioral post-test to assess changes in motor performance.

#### Data acquisition

Image acquisition was performed on a 1.5 T Philips MRI scanner (Best, The Netherlands) using an 8-channel SENSE head coil with a mirror for front-projected visual feedback. A T1-weighted anatomical image was acquired in the sagittal plane using 256 × 256 mm in-plane resolution, lasting approximately 5 min. The structural image was transformed to 1 mm^3^ voxel resolution and standard sagittal plane orientation by BrainVoyager QX v2.3 (Brain Innovation, Maastricht, The Netherlands). Functional images were acquired in 20 descending transverse plane slices using a gradient-echo T2^*^-weighted echo-planar image sequence with TR/TE of 2000/50 ms and a flip angle of 85°. The whole brain was covered using an in-plane resolution of 3.4 × 3.4 mm^2^ with 5 mm slice thickness and 1 mm gap width over a field of view of 220 × 220 mm^2^.

#### Functional localizer

The functional localizer was conducted to define the spatial extent of cM1. The functional localizer consisted of two conditions, *Rest* (16 s) and *Pinch* (30 s), where subjects were asked to relax or to firmly generate repetitive pinching, respectively. The instructed movement rate of 0.5 Hz was indicated by a color change of the instruction displayed on the screen. The functional localizer lasted approximately 4 min. Volumes were collected online from the Philips DRIN (Direct Reconstructor Interface) server and processed using fMRI analysis software (Turbo-BrainVoyager 3.0, TBV, Brain Innovation, The Netherlands). Functional data were obtained using a general linear model (GLM) employing head motion correction, coregistered with the anatomical image for precise localization of cM1. Our ROI was defined from active voxels (threshold of *t* > 3.0) within the hand-knob region (Yousry et al., 1997), anterior to the central sulcus (Figure 1D). This ROI, defined in the participant's native space, was subsequently used for the feedback signal throughout the neurofeedback training.

#### Baseline imagery

Following the localizer, baseline kMI was conducted to examine participants' abilities to activate cM1 during motor imagery. Both the scanning sequence and protocol of baseline imagery were identical to the functional localizer except that participants were instructed to perform only kMI of pinching. Specifically, subjects were asked to imagine performing the precision grip task. They were asked to focus on the motor and somatosensory aspects of the precision grip (Jeannerod, 1994). In other words, they were instructed to imagine performing pinching movements as done and felt during the functional localizer and familiarization of the force-matching task, but without overt movement.

#### Behavioral pre-test and post-test

The protocol of the behavioral task was the same as during familiarization. The only difference with the familiarization task was that only one horizontal bar was displayed, which had to be quickly and precisely reached by the isometric precision grip force (Figure 1C). Eight blocks of 10 trials were interleaved with 12 s periods of rest, each block lasting 33 s. The blocks, containing trials with only one of the two target force levels trained during the familiarization, were pseudo-randomly distributed among the runs.

#### Neurofeedback-guided motor imagery

The aim of the neurofeedback was to aid kMI of pinching toward an activation increase in cM1. Participants were instructed to alternately raise and lower the height of a continuously moving ball on the screen according to visual instructions, Imagine and Rest, respectively (Figure 1E). They were informed that the height of the ball represented the average activity in cM1 and that there was about a 5 s delay between their thoughts and the visual feedback. In order to control the ball, subjects were instructed to perform exclusively kMI of pinching during Imagine, without exerting any movement. During Rest, subjects were asked to focus on the sensation of breathing. Participants were given the same instructions as in baseline imagery with regards to the type of kMI to use to control the height of the ball. However, throughout the neurofeedback training they could change some aspects of the imagined pinching (i.e., pinching hard/soft pieces, and/or pinching faster). They were informed that the task was difficult and were asked to simply try their best and not become frustrated. The MR-compatible pinch sensor was used to monitor unintended movements.

Neurofeedback-guided imagery was organized in three 6-min runs. In each run, Rest and Imagine were presented for 16 and 30 s respectively, beginning with Rest. In total there were eight trials of each condition. In between runs, subjects were further verbally encouraged to perform at their best.

The neurofeedback signal was extracted from the ROI (i.e., cM1) online using Turbo-BrainVoyager. The signal was first smoothed using a three-point moving average and then subtracted from the average signal of the last five volumes of the previous Rest block (i.e., baseline), as described in our earlier work (Sulzer et al., 2013b). The signal was visually displayed such that a 2.5% increase in neurofeedback signal corresponded to the top of the screen.

### Data analysis

#### fMRI data processing and analysis

Preprocessing and statistical inference were performed using Brain Voyager QX 2.3. Head movements were calculated by spatial alignment of all volumes based on the first volume using trilinear/sinc interpolation. To remove non-linear drifts, a temporal high-pass filter of two cycles per time course was applied. Data were spatially smoothed using a Gaussian kernel with 6-mm full width at half maximum (FWHM). After preprocessing, the functional data were co-registered to the anatomical volume through a manual alignment of landmark points and transformed into Talairach space (Talairach and Tournoux, 1988).

A standard first-level general linear model (GLM) approach was applied in first-level analysis, with a design matrix including two regressors of interest (i.e., Imagery-Rest task and the unintended exerted force) and head movement regressors of no interest. The unintentionally exerted force regressor, used to monitor compliance to instructions not to move during motor imagery neurofeedback, was calculated from the down-sampled average force of the sensor in temporal windows of 2 s. In other words, involuntary muscle contractions during imagery were accounted for and excluded by regressing out the force in the GLM. Before preprocessing, the regressors were normalized to the interval [0, 1] and then convolved with a canonical hemodynamic response function (HRF). After normalization, the force regressor was orthogonalized to the motor imagery regressor, using Gram-Schmidt orthogonalization (Cheney and Kincaid, 2009). This procedure ensured that the parameter estimate of the task regressor was independent of any unintentionally exerted force.

#### ROI analysis

*Post-hoc* analysis was conducted on the Talairach-transformed functional cM1 ROI delineated for each subject in native space during the motor execution localizer. Statistical comparisons between the BOLD responses in the task were based on the fitted z-transformed and mean-corrected beta value extracted from the ROI. Beta values were used as the measure of cM1 activation, calculated as a single value representing the average activation over the entire run compared to baseline. Beta values represent the slope of the linear regression (or in other words, the magnitude of the relation) between the MI task and the cM1 BOLD signal. We examined any evidence of within-session neurofeedback learning, defined as a significant increase in beta values over runs, using One-Way repeated measures ANOVA (α ≤ 0.05).

#### Behavioral pre- and post-test analysis

**Figure 2:**
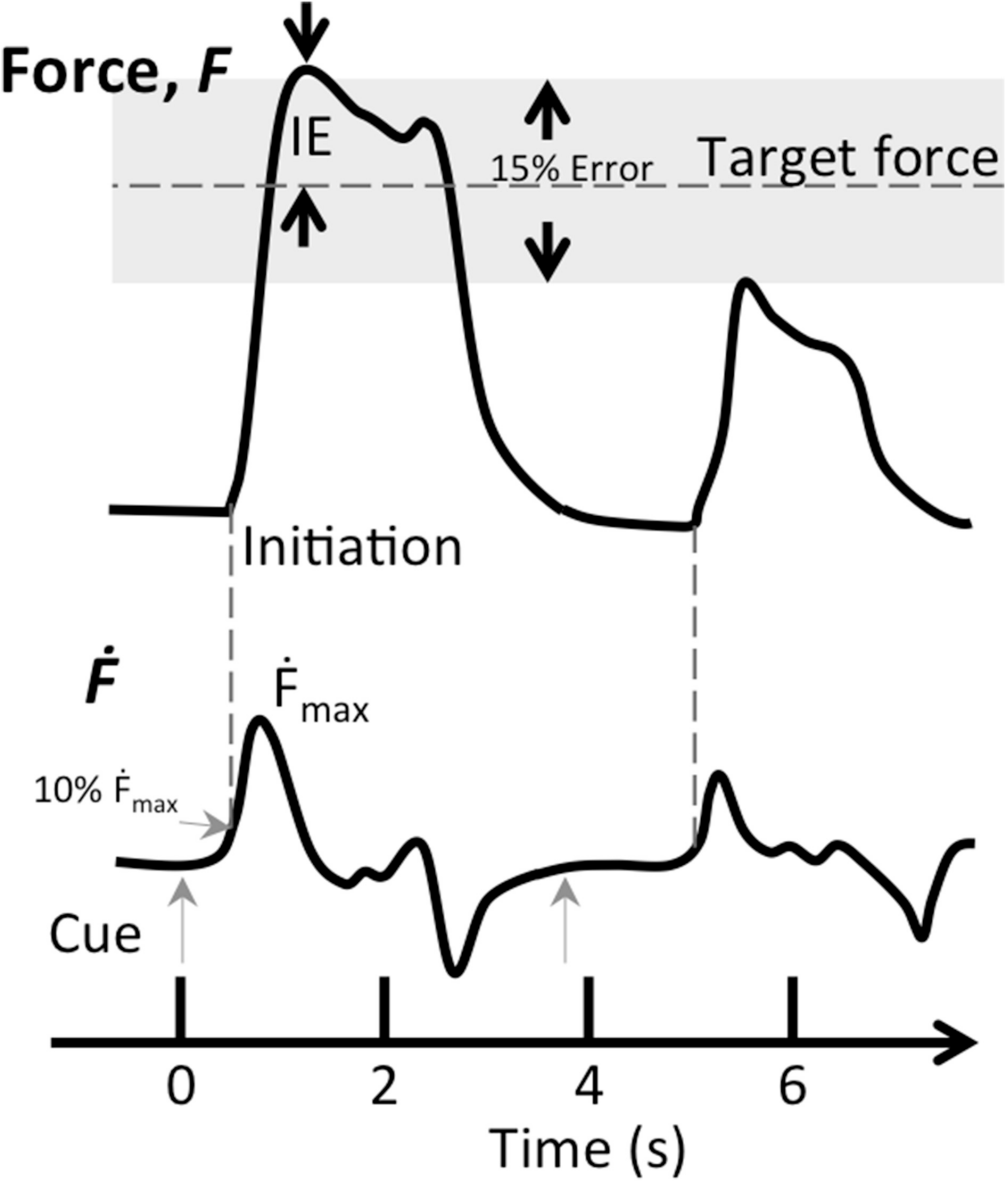
**Inclusion criteria for successful trials during the force matching task**. The two inclusion criteria for successful trials were target error (*IE*) being within 15% of target force (gray shaded area) and the first derivative of force (*Ḟ*) reaching 10% of maximum speed between 0.15 and 0.5 s after the visual cue (gray arrows). This figure shows two successive trials, the first trial (*t* = 0 s) fits both criteria, while the second one (*t* = 3.9 s) does not satisfy either criterion.

We analyzed behavioral data using Matlab R2012 (Mathworks, Natick, MA). A trial was considered successful when initiation (force derivative, *Ḟ*, above 10% of maximum) occurred between 150 and 500 ms from cue onset, representing the visuomotor delay to a cue. In addition, the applied force of a successful trial had to be within 15% of the target level, representing performance within three multiples of the trained accuracy (Figure 2). In each successful trial, we determined the accuracy, i.e., *Initial Error (IE)*, defined as the magnitude of the difference between the first local maximum after initiation and the target force level, divided by the target force level (see Figure 2 for graphical presentation of inclusion criteria). The *Maximum force derivative* (*Ḟ*_*max*_), corresponding to the speed of the vertical bar during isometric force contraction, was defined as maximum of the force derivative divided by the target force level. This quantity could also be thought of as jerk, however, for isometric contractions we consider force derivative to be more intuitive nomenclature. Changes in performance were evaluated by subtracting pre-test performance from post-test performance, normalized to pre-test performance (resulting in Δ*IE* and Δ*Ḟ_max_*),

(1) $$\Delta IE=\frac{IE_{post}-IE_{pre}}{IE_{pre}},\text{ and}$$

(2) $$\Delta {\dot{F}}_{max}=\frac{{\left({\dot{F}}_{max}\right)}_{post}-\;{\left({\dot{F}}_{max}\right)}_{pre}}{{\left({\dot{F}}_{max}\right)}_{pre}}.$$

Prior studies have shown that improvement in the speed-accuracy tradeoff indicates that motor skill acquisition is occurring (Willingham, 1998; Krakauer and Mazzoni, 2011). Thus, we defined a performance metric to take into account the contribution of the two normalized measures on the overall motor performance as:

(3) $$\Delta MP=\;\Delta {\dot{F}}_{max}-\;\Delta IE,$$

where Δ*MP* is the change in motor performance. Note that Δ*IE* is subtracted since a decrease in error is an improvement in accuracy. As such, this metric best represents the instructions to the participants, i.e., maximizing both speed and accuracy with equal deftness. Changes in performance outcomes were measured using a one-sample *t*-test (α ≤ 0.05). We used a first order regression analysis (α ≤ 0.05) to test whether cM1 beta values correlate with the outcome measures (Δ*MP*, Δ*IE*, and ∆*Ḟ*_*max*_). As a secondary outcome, we additionally performed an analysis of covariance (ANCOVA) on the differential relationships between cM1 beta values and Δ*IE, as well as* Δ*Ḟ_max_*; i.e., whether the modulation of one parameter outweighed the modulation of another. All statistics were performed using SPSS v19 (IBM, Armonk, NY).

#### Random effects (RFX) GLM group analysis

To identify the specificity of kMI on the whole brain, we examined activity in other regions using RFX group analysis. Standard second-level RFX analysis was conducted based on individual contrasts. Individual images were first applied in first-level contrasts and then combined in a summary statistic RFX GLM analysis. Images were percent-transformed and serially corrected, then corrected for multiple comparisons using cluster level correction at α < 0.05. Active regions were identified based on the nearest coordinate using a Talairach Daemon (Lancaster et al., 2000). We focused our analysis on motor and motor-related regions activated during motor imagery (Hétu et al., 2013).

#### Psychophysiological interaction (PPI) analysis

As our goal is to identify the role of cM1 in a force control task, we must also account for the possibility that cM1 may interact with other regions in the sensorimotor network (Kasess et al., 2008; Guillot et al., 2012). Therefore, we conducted a PPI analysis to examine whether there was any evidence of such interactions. PPI analysis (Friston et al., 1997) is a measure of effective connectivity developed in order to determine whether a psychological variable, such as kMI, modulates the connectivity between physiological variables, i.e., brain regions. First, the time course of the BOLD signal of the cM1 hand region was extracted for each subject. Then a PPI regressor, which is the dot product of the time course and the HRF-convolved regressor, was created and mean corrected. The design matrix thus included the PPI regressor, the mean-corrected time course, the mean-corrected task regressor convolved with the HRF, the ortho-normalized force, head movement regressors and a constant. We then repeated the RFX analysis with the PPI regressor, as described above.

## Results

### ROI location and analysis

**Figure 3:**
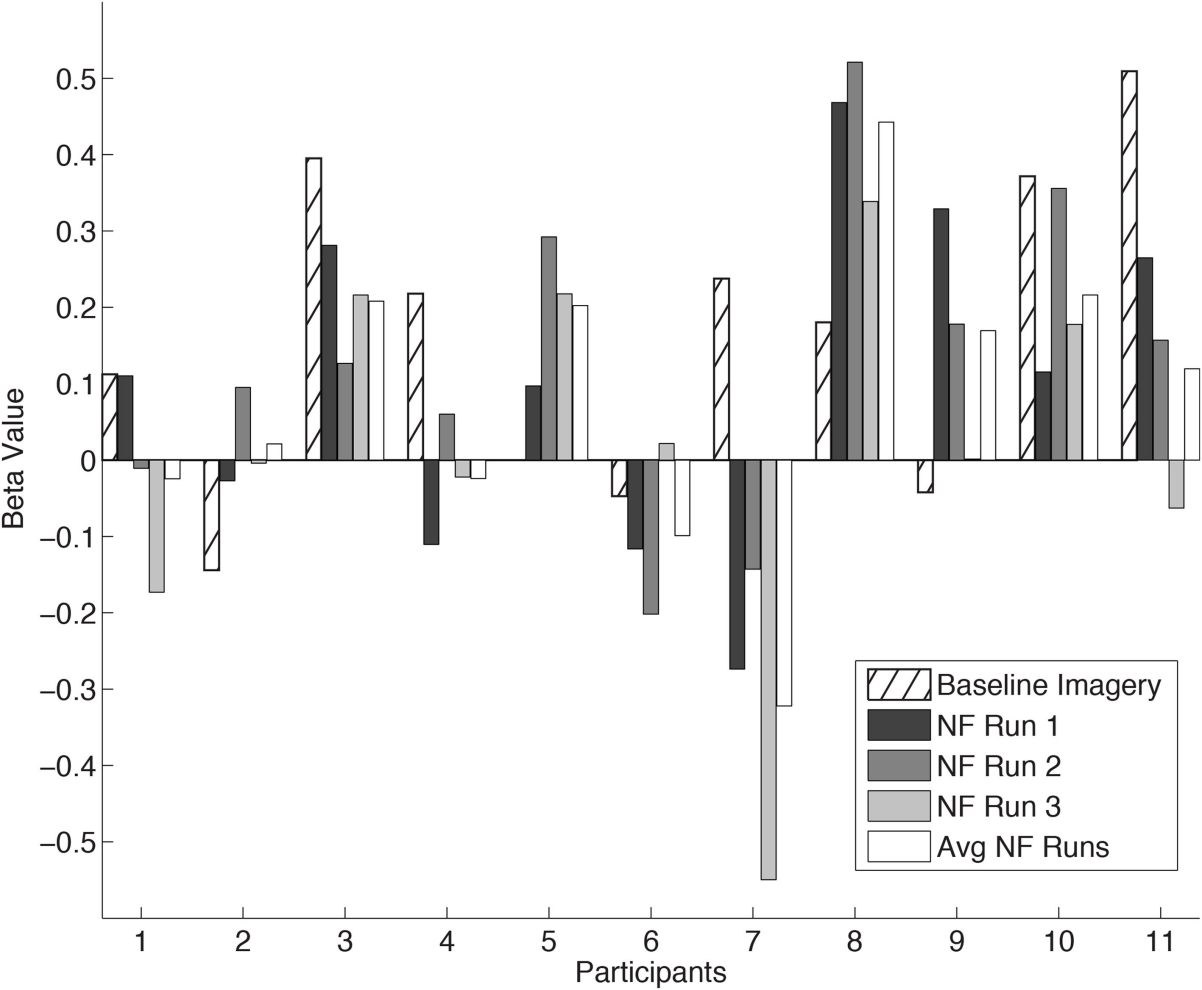
**cM1 beta values in all eleven Participants (P1… P11)**. Individual cM1 beta values during baseline imagery, the three neurofeedback runs (NF run1, run2, run3) and the average across runs (Avg NF runs).

The mean coordinates of the ROI center for the hand region in Talairach space across participants, located anteriorly to the central sulcus, was *x* = −35 ± 5.1; *y* = −24 ± 4.6; *z* = 51 ± 2.9. The individual ROI beta values are presented in Figure 3 for all the participants. Two participants were excluded from this and subsequent analysis due to malfunction of the force sensor and misalignment of target ROIs, respectively. In one participant (P5) the baseline imagery beta value was not measured due to a failure in extracting the unintentionally exerted force regressor. The remaining 11 participants showed a large variation in ability to self-regulate cM1 using neurofeedback as hypothesized. The average cM1 activity over all neurofeedback runs was positive for most participants [*t*-test, *t*_(10)_ = 1.35, *p* = 0.20]. In general, cM1 activity during neurofeedback was lower than during baseline imagery, but the difference was not statistically significant [paired *t*-test, mean difference = −0.08, *t*_(10)_ = −0.99, *p* = 0.34]. One-Way repeated measures ANOVA revealed no within-session changes in cM1 self-regulation [*F*_(1)_ = 1.97; *p* = 0.19].

### Behavioral pre- and post-test analysis

As a group, no significant changes in motor performance were found between pre- and post-tests. One-sample *t*-tests did not reveal any significant differences in ∆*Ḟ*_*max*_ [*t*_(10)_ = 1.91, *p* = 0.08] or for Δ*IE* [*t*_(10)_ = −0.05, *p* = 0.95]. In pre-test, 18 ± 13% (mean ± SD) of trials were dropped, and in post-test 15 ± 11% of trials were dropped, as they did not fulfill the criteria for successful trials.

### Correlation of M1 with changes in motor performance

**Figure 4:**
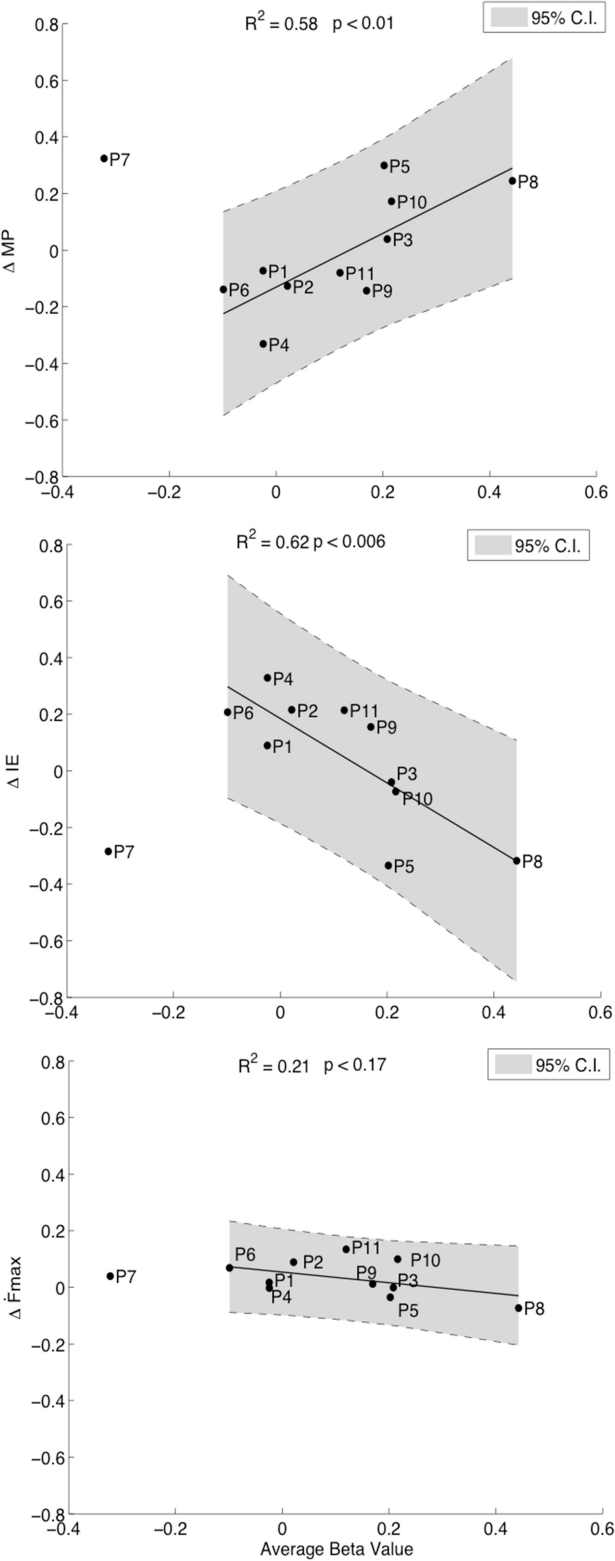
**Correlations of cM1 up-regulation with behavioral outcome measures**. Correlation of normalized cM1 beta values during neurofeedback-guided motor imagery with an overall improvement in motor performance (Δ*MP*, top), with a decrease in initial error (Δ*IE*, inverse of accuracy, middle) and a decreasing trend in maximum first force derivative (Δ*Ḟ_max_*, speed of moving bar, bottom).

Our hypothesis was that the degree of cM1 activity during kMI guided by neurofeedback would be related to improvements in motor performance. First order regression analyses revealed positive correlations between cM1 beta values over all runs and improvements in motor performance, *ΔMP* (*R*^2^ = 0.58, *p* = 0.01, Figure 4, top). This relation was driven by a statistically significant improvement in accuracy, i.e., decrease in Δ*IE* (*R*^2^ = 0.62, *p* < 0.006, Figure 4, middle) with an insignificant decrease in speed, ∆*Ḟ*_*max*_ (*R*^2^ = 0.21, *p* = 0.17, Figure 4, bottom). The increase in accuracy with cM1 beta outweighed the decrease in speed (ANCOVA, *F*_(1)_ = 15.59, *p* = 0.0012). In these correlations, P7 was identified as an outlier and removed from analysis, as it was consistently outside the 95% CI of each correlation (Figure 4). We validated that no other data point was outside the 95% CI using ten-fold cross-validation analysis of all other combinations (*N* = 11 − 1) of data points. Re-evaluating the behavioral pre- and post-test analysis after removing this outlier did not significantly change the results: ∆*Ḟ*_*max*_ [*t*_(9)_ = 0.09, *p* = 0.11] or for Δ*IE* [t_(9)_ = 0.001, *p* = 0.78].

The relation between cM1 and motor performance was not explainable with VMIQ scores, which correlated neither with *ΔMP* (*R*^2^ = 0.001, *p* = 0.93) nor with cM1 beta values (*R*^2^ = 0.04, *p* = 0.54).

### RFX GLM group analysis

**Figure 5:**
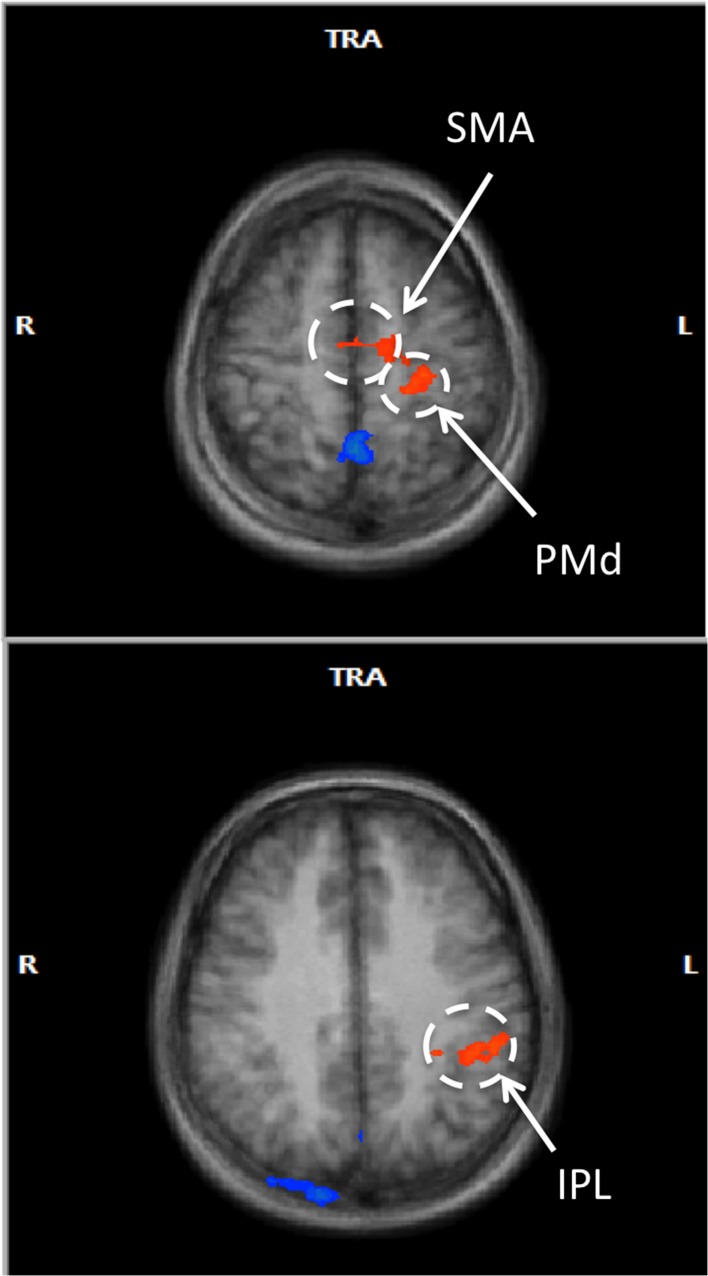
**Voxel-wise RFX analysis of neurofeedback-guided motor imagery**. Above, frontal lobe (*z* = 55 mm), below, inferior parietal lobule (*z* = 34 mm). Radiological convention (contralateral/left is on right). Cluster level corrected, *p* < 0.05. Orange: BOLD signal increase; blue: BOLD signal decrease.

In a voxel-wise analysis, we investigated whether other brain regions were activated during the neurofeedback-guided motor imagery as a measure of specificity. Due to the small number of subjects (*N* = 10), a cluster-level correction for multiple comparisons was applied. The active regions are listed in Table 1 and illustrated in Figure 5. Positively activated regions were centered in the contralateral medial frontal gyrus, including SMA and dorsal premotor region (PMd), putamen, caudate, as well as in the inferior parietal lobule (IPL) and sub-gyral region. Negatively activated regions included ipsilateral middle temporal and frontal gyrus, precuneus, insula, paracentral lobule and contralateral middle occipital gyrus.

**Table 1:** **Center of gravity of the positively and negatively activated regions in the subjects with positive M1 beta values during motor imagery**.

	**Side**	**Tailarach XYZ**			**Voxels**	***T*-value**
**POSITIVELY ACTIVATED REGIONS**						
Medial frontal gyrus	L	−10	−7	55	1848	10.25
Inferior parietal lobule	L	−49	−34	34	4088	9.40
Caudate	L	25	−37	8	2352	8.64
Putamen	L	−25	−4	9	3912	13.35
Subgyral region	L	−21	29	9	3896	7.43
**NEGATIVELY ACTIVATED REGIONS**						
Middle temporal gyrus	R	40	−69	−24	9176	−7.69
Middle frontal gyrus	R	32	10	49	1768	−7.80
Insula	R	34	−16	18	944	−8.16
Middle occipital gyrus	L	−31	−84	18	5424	−9.25
Paracentral lobule	R	1	−38	50	6152	−10.51
Precuneus	R	5	−56	39	2248	−8.07

*Cluster-level corrected p-values, all p < 0.0001, voxel size of 1 mm^3^*.

### *Post-hoc* analysis of correlations with other regions

Additional *post-hoc* analyses were performed to see whether the significant correlations of M1 BOLD signal with outcome measures were unique to cM1, or were perhaps a general effect existing over the motor imagery network. To represent this active network, beta values were extracted from the two positively activated clusters revealed by the RFX GLM group analysis (SMA/PMd and IPL). These values were fed into a linear mixed model (SPSS, Armonk, NY) as covariates, including cM1 beta values as the independent variable and *ΔMP* as the dependent variable. Accounting for this covariation, cM1 activation maintained its significant linear relationship with *ΔMP* [*t*_(3)_ = 3.20 *p* < 0.01]. There were no significant correlations of *ΔMP* with BOLD signals in SMA/PMd [*t*_(3)_ = 1.22, *p* = 0.26] and IPL [*t*_(3)_ = −1.21, *p* = 0.27].

### PPI analysis

PPI RFX group analysis did not reveal any significant interactions between cM1 and other regions during MI. However, as it is likely that the influence of these regions may vary with the ability to activate cM1, a *post-hoc* ROI correlation analysis on SMA/PMd and IPL was conducted. No significant correlations were found between cM1 beta and SMA/PMd (*R* = 0.29, *p* = 0.40) and IPL beta (*R* = 0.52, *p* = 0.12) beta values.

## Discussion

Motor imagery is an established method of supporting motor learning and its neural mechanisms are well known; yet it remains an open question regarding how these mechanisms translate to motor improvements. Here, we attempted to use M1 activity as the independent variable during kMI via rtfMRI neurofeedback, predicting that greater M1 activation would lead to performance improvements in a simple motor task. We found correlations between cM1 activation and performance changes in an isometric force precision grip task. Such correlations were not found in other regions activated during kinesthetic MI (i.e., SMA, PMd, or IPL). These data strongly suggest that cM1 is primarily involved in the beneficial effects of motor imagery.

While much is known regarding the neural correlates of motor imagery (for recent review, see Hétu et al., 2013), there is surprisingly sparse evidence relating these data to motor performance. Similarly, there are several studies that show self-regulation of sensorimotor areas using rtfMRI, but only a few relate this self-regulation to motor performance as we have pursued in this study. In a well-controlled study using rtfMRI neurofeedback, Bray et al. (2007) reported that participants were able to self-regulate the BOLD signal in primary sensorimotor cortex using instrumental conditioning with a displayed reward feedback (dollar bill) when the BOLD signal change increased over a threshold during motor imagery. In addition, they found that over four conditioning blocks within a single session, reaction times in pressing a button significantly improved. Subramanian et al. used feedback of SMA BOLD signal in five Parkinson's patients during motor imagery, finding increased motor speed in finger tapping (Subramanian et al., 2011). Both of these reports show that SMA and M1 are involved in the beneficial effects of MI, but they do not explore the possibility of modulation from other brain regions. In contrast, Zhao et al. reported improvements in the execution time of a motor sequence following successful self-regulation of PMd (Zhao et al., 2013). These studies used simple models to find the association between regulation and behavioral change (i.e., ability to modulate results in performance improvement). In contrast, we applied a specific kMI strategy (i.e., pinching) and found a more descriptive linear relationship between cM1 activation during kinesthetic motor imagery and motor performance changes, demonstrating a functional relationship. This correlation sheds light on how much modulation is needed to facilitate a behavioral change.

One interpretation of the correlation between the induced increase in BOLD signal and motor performance is that the endogenous stimulation of cM1 by means of kMI neurofeedback enabled skill improvement. This interpretation may support results from earlier studies using exogenous cM1 stimulation in the form of TMS to enhance mental rotation performed by visual or motor imagery (Tomasino et al., 2005; Bode et al., 2007). However, a recent meta-analysis of task-related activations during learning questions whether M1 is the primary region or simply downstream of correlated changes occurring in higher order regions, such as PMd (Hardwick et al., 2013). Our data reveal correlations between behavioral changes and cM1 activation during neurofeedback-guided motor imagery. We additionally accounted for specificity of the role of cM1 within the motor imagery network by including activation of SMA, PMd, and IPL in our regression analysis. Therefore, it seems that, at least during kinesthetic motor imagery, cM1 activation could have a leading role in changes in motor performance, probably due to repeated and enhanced activation of cM1.

An alternate interpretation of the correlation between changes in performance and cM1 is that subjects able to up-regulate cM1 are also more likely to improve their motor performance. In other words, the two quantities are associated, but without any direct causal relationship. Our experimental design cannot confirm this interpretation, but if true, the data would indicate that cM1 activity is an important biomarker to identify candidates for neurofeedback-guided MI training. We are unable to compare this potential biomarker to other MI biomarkers, such as skin conductance response or chronometric measures of imagery (Guillot et al., 2008), as they were not included in our investigation.

It is interesting to note that cM1 activity correlated positively with accuracy, but not speed. This is consistent with studies that show improvements in accuracy in early stages of learning (Hikosaka et al., 2002). However it would be unexpected that M1 would be driving this change, as the early stage is driven by associative and sensorimotor regions (Lehéricy et al., 2005). While cM1 is not an associative region, the activity measured was during kMI, not during the task, as the aforementioned studies examined. It may be possible that sensorimotor areas such as M1 have differential modulatory effects on motor performance depending on the conditions of their activation, i.e., during MI or execution (Karni et al., 1995; Lotze et al., 2003).

We also found that motor performance decreased in those participants with low cM1 activity during neurofeedback-guided MI (Figure 4, top). Such a result may suggest that low cM1 activation during motor imagery is detrimental to motor performance. While the negative bias of the linear model may initially seem counterintuitive, such decrements are in fact expected for high performance tasks where sustained attention is required over a long period of time, (Mackworth, 1968; Robertson et al., 1997). On the other hand, it is also possible that the low performance during neurofeedback-guided imagery had discouraged subjects in the following post-test. While we acknowledge this possibility, we continuously encouraged participants during the experiment to prevent frustration.

In this study we used neurofeedback as a tool to help subjects focus their kMI specifically on cM1, with the intent of inducing higher levels of activity in this target region than through imagery alone. Instead, we found no significant improvement, but more likely a decrement, when comparing baseline imagery without neurofeedback to neurofeedback performance (Figure 3). Yet, baseline imagery was only a single 4-min run, a difficult comparison to the average of three 6.5-min neurofeedback runs. However tenuous the comparison, the lack of improvement of M1 over time could be due to divided attention of the neurofeedback and imagery (Pashler, 2000). Such divided attention has been avoided in other sensorimotor rtfMRI neurofeedback studies by using terminal feedback (Bray et al., 2007; Johnson et al., 2012). Yet we have no evidence to suggest that the mixture of externally- and internally directed cognition play a role in modulation of M1 as no prefrontal areas were significantly activated (Dixon et al., 2014). The variability of M1 activity may also be a typical consequence of motor imagery ability (Lotze and Halsband, 2006; Sharma et al., 2006; Munzert et al., 2009; Madan and Singhal, 2012), and would be consistent with other work in neurofeedback (Berman et al., 2012). Indeed, a decrement could also be imposed by habituation (Rankin et al., 2009) a phenomenon that has shown to play a role in other neurofeedback studies (Sulzer et al., 2013b; Greer et al., 2014). It is important to note that our goal was not to evaluate the level of success of neurofeedback performance, but rather its potential as a method to support endogenous cM1 regulation.

Quite often, the benefits of motor imagery on motor performance were attributed to the individual's ability to produce vivid movement-related mental imagery (Munroe et al., 2000). Although we could not systematically measure the vividness of imagery at the end of each cM1 modulation block during neurofeedback, participants assured their compliance to instructions (i.e., kinesthetic motor imagery) at the end of the experiment. Activation in motor areas, especially within a parieto-premotor network, was parametrically linked to imagery vividness (Lorey et al., 2011). In our data, the relation between cM1 and motor performance was not explainable with VMIQ scores. Most likely, although a self-report questionnaire such as the VMIQ has led to valid and useful results for measuring motor imagery ability, the results are always affected by a strong subjectivity component (Guillot and Collet, 2005). Few participants reported freely that some items were rated with high score (i.e., low imagery ability) due to their poor level of motor expertise in the task to be imagined (i.e., if they have never performed a task).

While MI training has been found helpful in neurologically healthy subjects, its inconsistent effectiveness in neurorehabilitation has perplexed researchers (for review see Malouin and Richards, 2013). For instance, a number of randomized controlled trials have shown large improvements in clinical outcome scores with MI training (Liu et al., 2004; Page et al., 2005, 2007, 2009; Braun et al., 2006), but others (Bovend'Eerdt et al., 2010; Ietswaart et al., 2011) revealed negative results. Aside from inter-study differences such as the type and amount of physical practice, specificity and impairment level, it is additionally difficult to evaluate how well the MI was performed. Our data showing variable cM1 activity at the individual level could account for the variance found between subjects and between studies. Additionally, rtfMRI could be used to quickly identify those patients who may most profit from MI therapy. However, it should also be noted that the neurological injury itself may also contribute toward MI ability (Di Rienzo et al., 2014), and therefore the application of this conclusion toward impaired neurological models is speculative.

## Conclusions

The purpose of this study was to identify whether and how cM1 activation during kinesthetic MI affects motor performance in a precision grip task. We provide compelling evidence that cM1 BOLD activity during imagery predicts improvements in motor performance. These data suggest that the ability to activate M1 through motor imagery may play a key role in determining the effectiveness of imagery training. This study introduces a novel approach toward *endogenous* stimulation for the purpose of neurophysiological investigation.

### Conflict of interest statement

The authors declare that the research was conducted in the absence of any commercial or financial relationships that could be construed as a potential conflict of interest.
